# Recurrent Pericarditis in a Middle-Aged Female with MEFV Mutation

**DOI:** 10.3390/jcdd13050218

**Published:** 2026-05-19

**Authors:** Xiaohang Liu, Tongxin Xiao, Lihua Zhang, Zhongjie Fan, Xinglin Yang, Zhuang Tian

**Affiliations:** 1 Department of Cardiology, Peking Union Medical College Hospital, Chinese Academy of Medical Sciences and Peking Union Medical College, Beijing 100006, China; 2 Department of Internal Medicine, Peking Union Medical College Hospital, Chinese Academy of Medical Sciences and Peking Union Medical College, Beijing 100006, China; 3 Department of Endocrine, Peking Union Medical College Hospital, Chinese Academy of Medical Sciences and Peking Union Medical College, Beijing 100006, China

**Keywords:** recurrent pericarditis, *MEFV* gene, autoinflammation, tuberculosis

## Abstract

Recurrent pericarditis (RP) remains challenging, especially in tuberculosis (TB)-endemic regions where empirical anti-TB therapy is often unnecessarily prolonged. We report a 35-year-old woman with three RP episodes over six months, presenting with pleuritic chest pain, elevated inflammatory markers, and moderate-to-large pericardial effusion. Extensive infectious (including TB), autoimmune, and malignancy workups were negative. Cardiac magnetic resonance revealed persistent pericardial late gadolinium enhancement despite clinical remission. Whole-exome sequencing identified a heterozygous *MEFV* c.442G>C (p.Glu148Gln) variant, suggesting an autoinflammatory predisposition. Although the patient finally achieved sustained symptom-free status for six months on a standardized low-dose colchicine regimen, still over 10% of patients have recurrent symptoms receiving colchicine in addition to conventional anti-inflammatory therapy with aspirin or ibuprofen. This case highlights the shifting paradigm from an infection-centered to an autoinflammatory framework for RP in TB-endemic countries, underscores the role of *MEFV* variants in idiopathic recurrent pericarditis, and illustrates the real-world gap between genetic insights and therapeutic accessibility to IL-1 inhibitors in resource-limited settings.

## 1. Introduction

Recurrent pericarditis (RP) is defined as a recurrence of pericarditis after a symptom-free interval of at least 4 to 6 weeks following an initial acute episode. It affects approximately 15–30% of patients after acute pericarditis, with up to 50% experiencing multiple recurrences, leading to significant morbidity and impaired quality of life. Diagnosis of recurrence is established according to the same criteria as those used for acute pericarditis [1,2]. A simple aetiological classification of pericardial diseases divides them into infectious and non-infectious causes. The aetiology is diverse and depends on factors such as the epidemiological background, patient population, and clinical setting [1]. Unlike in developed countries, traditionally, in countries with a high burden of tuberculosis (TB), including China, recurrent or unexplained pericardial effusion has long been empirically attributed to tuberculous pericarditis. As a result, diagnostic anti-tuberculosis therapy is frequently initiated once recurrence is observed, even in the absence of microbiological or pathological confirmation [3]. However, the etiologic landscape of pericardial disease has been undergoing a significant transition. With socioeconomic development and improved infection control, the proportion of infection-related pericarditis has gradually declined, while immune-mediated and autoinflammatory mechanisms are increasingly recognized as dominant drivers of idiopathic recurrent pericarditis (IRP) [3,4,5].

The management of RP is often challenging. First-line therapies include nonsteroidal anti-inflammatory drugs (NSAIDs) and colchicine, but a substantial proportion of patients become steroid-dependent or refractory to conventional treatments [6,7]. Recent advances have highlighted the central role of interleukin-1 (IL-1) in the pathogenesis of IRP, leading to the successful use of IL-1 inhibitors such as anakinra and rilonacept in colchicine-resistant and corticosteroid-dependent cases [8,9,10]. Meanwhile, this epidemiologic shift has created a growing clinical paradox in China: despite mounting evidence supporting IRP as an IL-1-driven autoinflammatory disorder, many patients still receive prolonged empirical anti-TB therapy or high-dose corticosteroids, exposing them to unnecessary toxicity while delaying appropriate immunomodulatory treatment.

In parallel, genetic predisposition has emerged as an important contributor to autoinflammatory phenotypes of pericarditis. Variants in the *MEFV* gene, classically associated with familial Mediterranean fever (FMF), have been linked to dominant or recessive autoinflammatory phenotypes, including pericarditis. Such variants have been increasingly reported in patients with RP, even in the absence of typical FMF manifestations [11,12].

In this report, we describe a case of recurrent pericarditis in a 35-year-old woman carrying a heterozygous *MEFV* p.Glu148Gln variant. This case potentially illustrates the evolving disease spectrum of pericarditis in contemporary China and highlights the diagnostic and therapeutic challenges in the transition from empiric, experience-based treatment toward mechanism-driven precision diagnosis and therapy.

## 2. Case Presentation

A 35-year-old woman presented with recurrent pleuritic chest and back pain for 6 months, accompanied by mild dyspnea and low-grade fever (maximum temperature 37.8 °C). The initial episode was mild and resolved within 2 days with a single 300 mg dose of ibuprofen. After that, she experienced three relapses, each lasting several days, with symptom-free intervals of approximately 8, 3, and 6 weeks, respectively (Table 1). During symptomatic periods, inflammatory markers were markedly elevated (Table 1; C-reactive protein [CRP] 247.4 mg/L, and interleukin-6 [IL-6] 350 pg/mL at her first recurrence).

At her first in-hospital evaluation during an active flare, electrocardiogram demonstrated sinus tachycardia with suspected PR-segment depression. Echocardiography revealed moderate-to-large pericardial effusion, with maximal depth increasing from 18.7 mm to 23.5 mm on repeat examination, accompanied by partial respiratory collapse of the inferior vena cava. Coronary computed tomography (CT) angiography showed no significant coronary stenosis. An extensive infectious evaluation did not identify a causative pathogen, including serum metagenomic next-generation sequencing (mNGS). Tuberculosis was repeatedly evaluated: two interferon-γ release assays (T-SPOT.TB) and one purified protein derivative (PPD) skin test performed during prior flares were all negative. In terms of treatment response, after the first recurrence, she recovered on ibuprofen 300 mg twice daily plus colchicine 0.5 mg once daily, and a pericardiocentesis (approximately 15 mL of serous fluid, which was unfortunately not sent for biochemical or microbiological testing) at a local hospital. However, the second and third recurrences occurred during periods of intermittent maintenance therapy with lower doses of ibuprofen and colchicine (Table 1). Specifically, the second recurrence was managed by increasing ibuprofen to 600 mg every 8 h and adding prednisone 20 mg daily. The third recurrence was subsequently controlled by resuming ibuprofen 200 mg twice daily and prednisone, which was then gradually tapered.

**Figure 1:**
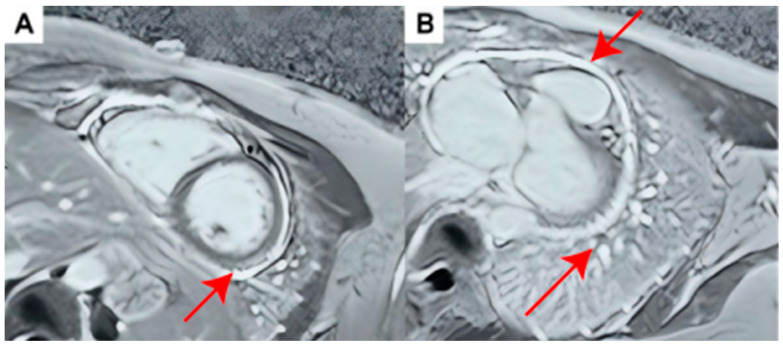
Cardiac Magnetic Resonance Imaging Findings. CMR was performed approximately 2 weeks after the most recent symptomatic flare, when her symptoms had improved, and CRP was within the normal range. Short-axis late gadolinium enhancement images. (**A**) Subepicardial delayed enhancement (red arrow) of the inferior and lateral walls of the left ventricle; (**B**) Extensive pericardial thickening with delayed enhancement (red arrow).

When she was admitted to our hospital for further evaluation, she had been asymptomatic for about three weeks while receiving ibuprofen 200 mg twice daily, colchicine 0.5 mg once daily (later increased to 0.5 mg twice daily), and prednisone 2.5 mg daily. Physical examination showed no edema. Routine laboratory tests, cardiac enzymes, and NT-proBNP (117 pg/mL) were within normal ranges. Inflammatory markers were suppressed during remission (CRP < 0.5 mg/L, serum amyloid A [SAA] 2 mg/L), and serum interleukins (IL-6, IL-8, IL-10) were negative. Autoimmune screening revealed a low-titer positive antinuclear antibody [ANA] (1:80), while the antinuclear antibody panel, complement levels, immunoglobulins, and antiphospholipid, rheumatoid, and anti-neutrophil cytoplasmix antibody (ANCA) panels were all negative. Repeat infectious evaluation, including a repeat blood T-SPOT.TB test, rubella virus, cytomegalovirus, and Coxsackie virus remained negative. Comprehensive screening for occult malignancy, including tumor markers and breast/abdominal ultrasonography, was negative. Antecubital venous pressure was measured at 6.5 cmH_2_O. Echocardiography showed normal left ventricular function (ejection fraction 70%) with no pericardial effusion. However, it revealed enhanced echoes and mild localized pericardial adhesion along the lateral and posterior walls of the left ventricle. Hemodynamic assessment for constrictive physiology was negative, with a mitral E-wave inspiratory variation of 14%, a normal E/A ratio of 1.0, and a mean E/e’ of 6. The inferior vena cava measured 9 mm with normal respiratory collapse. Crucially, a retrospective review of her prior cardiac magnetic resonance (CMR) study, obtained during clinical remission 2 weeks after the most recent symptomatic flare when her symptoms had improved and CRP returned to normal range (Figure 1), revealed diffuse pericardial thickening with prominent late gadolinium enhancement (LGE), particularly over the right ventricle. This finding demonstrated persistent localized pericardial inflammation despite clinical remission and normal inflammatory markers, thereby explaining the relapsing course during medication tapering. Because she had experienced multiple relapses despite more than three months of first-line therapy (NSAIDs plus colchicine), whole-exome sequencing (WES) was pursued to explore a potential autoinflammatory predisposition. WES identified a heterozygous *MEFV* c.442G>C (p.Glu148Gln) variant.

A diagnosis of recurrent pericarditis, presumably idiopathic, was established after the exclusion of known secondary etiologies. The patient was started on a standardized regimen of colchicine 0.5 mg once daily for a minimum of 6 months, with advice for close surveillance for potential constrictive physiology. At the 6-month follow-up, she remained symptom-free with persistently normal inflammatory markers.

## 3. Discussion

This case describes a 35-year-old woman with RP [1,13]. Although one flare occurred after a symptom-free interval of 3 weeks and therefore did not strictly meet the conventional 4–6 weeks criterion for recurrence, the other two flares occurred after intervals of 8 and 6 weeks, respectively. Thus, the overall clinical course fulfilled the diagnosis of RP, while the short-interval flare may represent an early relapse or incomplete remission. Extensive workup excluded common infection (including tuberculosis and coronavirus disease-19 [COVID-19]-related), connective tissue diseases, and neoplastic causes. Cardiac multimodality imaging was pivotal in assessing pericardial inflammation and ruling out constrictive physiology [14,15]. Thus, the patient’s presentation aligns with IRP, a diagnosis of exclusion often driven by autoinflammatory pathways [5,16]. Notably, whole-exome sequencing revealed a heterozygous variant in the *MEFV* gene (c.442G>C, p.Glu148Gln).

At the mechanistic level, recurrent pericarditis is now increasingly understood as an autoinflammatory disease characterized by dysregulation of innate immunity [3,4]. Central to this process is IL-1, a master cytokine that orchestrates inflammatory amplification within the pericardium. Experimental and clinical studies have consistently demonstrated excessive IL-1 signaling in patients with IRP, providing the biological basis for IL-1–targeted therapies [4,5]. The *MEFV* gene encodes pyrin, a key regulator of inflammasome activation. Under physiological conditions, pyrin modulates inflammasome assembly and prevents inappropriate activation of the NLRP3 inflammasome. Pathogenic *MEFV* variants may impair this regulatory function, leading to uncontrolled inflammasome activation, excessive caspase-1 signaling, and increased production of IL-1β [11,12,13,17,18]. Currently, more than 300 variants in the *MEFV* gene associated with FMF have been reported. There is still no consensus on whether the E148Q variant identified in our patient is pathogenic or non-pathogenic [19]. It was reported that the allele frequency of pyrin E148Q was 15% among Chinese healthy controls [20] and increased to 22.7% in Chinese FMF patients [19]. The E148Q variant by itself may not suffice to trigger FMF, but in some carriers of this low-penetrance variant, the disease might emerge following exposure to unidentified environmental or additional genetic factors [19]. This mechanism provides a plausible explanation for recurrent, self-sustaining pericardial inflammation even in the absence of identifiable triggers.

For RP treatment, according to the 2015 European Society of Cardiology guidelines, the standard management algorithm for recurrent pericarditis recommends NSAIDs and colchicine as first-line therapy, with corticosteroids reserved for patients who do not respond to NSAIDs. Colchicine is always used as an adjunct to NSAIDs or corticosteroids. In more refractory cases, triple therapy combining NSAIDs, colchicine, and corticosteroids may be considered [1]. More recently, expert opinion lacking support from formal trials or prospective studies recommended that, following the failure of NSAIDs and colchicine, patients with an inflammatory phenotype should receive an anti-IL-1 agent, either as monotherapy or in combination with colchicine. In contrast, patients without an inflammatory phenotype are better suited for corticosteroids [21]. Overall, colchicine remains a cornerstone, reducing recurrence rates from ~32% to 11% [6,22]. However, our patient relapsed despite a 3-month course, indicating a possible colchicine resistance, although it could not be confirmed because these relapses occurred during dose reduction. A definite colchicine resistance should be distinguished from relapse during colchicine dose reduction or irregular medication use. The definition of colchicine resistance is mainly derived from FMF and remains debatable, and it is generally uncommon, reported in approximately 5–10% FMF patients [23]. Some *MEFV* genotypes, especially homozygous M694V [24], have been associated with lower colchicine response and a higher inflammatory burden. Although the E148Q variant in our case is currently classified as a variant of uncertain significance, it would be interpreted as a possible marker of autoinflammatory susceptibility rather than evidence of genetically determined colchicine resistance.

While our patient did not fulfill diagnostic criteria for FMF and colchicine resistance, the genetic finding suggests a predisposition to innate immune dysregulation. This may explain her incomplete response to colchicine alone, as colchicine primarily suppresses neutrophil function but may not fully block IL-1 signaling in genetically primed individuals. From a pathophysiological standpoint, IL-1 inhibition would therefore represent a rational escalation strategy [8,9,25]. Recent RHAPSODY and AIRTRIP trials demonstrated that IL-1 inhibitors like rilonacept and anakinra significantly reduce recurrence and enable steroid tapering in refractory RP [9,10]. However, it was not initiated in this patient considering drug affordability and accessibility. It indicates another aspect of this case lies at the intersection of precision medicine and socioeconomic reality. In real-world clinical practice in China and other developing countries, IL-1 inhibitors remain largely inaccessible due to cost, regulatory barriers, or limited insurance coverage. Despite these constraints, the patient has remained symptom-free for six months on a standardized 0.5 mg daily dose of colchicine. This clinical observation suggests that in certain individuals with a potential autoinflammatory background, the consistency of anti-inflammatory therapy might be as critical as the absolute dosage. The success of the current regimen, contrasted with her prior relapses during periods of intermittent dosing, underscores the possibility that maintaining a steady anti-inflammatory environment is essential to prevent the activation of innate immune pathways—especially when a genetic predisposition is suspected. Consequently, clinicians and patients may have to accept suboptimal treatment strategies despite having identified a potential pathogenic target. If future relapses occur despite optimized colchicine therapy, IL-1 inhibition would be a rational strategy when available.

This case also prompts critical reflection on entrenched diagnostic habits. In TB-endemic or previously high-burden regions, recurrent pericardial effusion is frequently equated with tuberculous pericarditis, leading to empiric anti-TB therapy once recurrence occurs. Although TB should never be overlooked and such an approach was historically justified, it is increasingly misaligned with current disease patterns, and empirical anti-tuberculosis treatment carries substantial risks [2,3]. This diagnostic inertia is not limited to China but extends to many developing and middle-income countries experiencing similar epidemiologic transitions.

Given the possible genetic predisposition identified in this case, a long-term management strategy remains paramount. Should the disease recur despite standardized first-line therapy, a structured escalation would be warranted. While IL-1 inhibitors represent a rational targeted approach, their limited accessibility often necessitates the use of traditional immunosuppressants as steroid-sparing agents. In such scenarios, the addition of azathioprine or methotrexate could be considered to achieve deeper inflammatory control and facilitate corticosteroid withdrawal.

This case also has several limitations. First, the exclusion of rare infiltrative, histiocytic etiologies, in particular, Erdheim-Chester disease (ECD), is incomplete. ECD is a rare non-Langerhans histiocytosis increasingly recognized as a potential cause of pericardial and myocardial involvement. It may present with recurrent pericarditis and pericardial effusion, even in the absence of classic systemic manifestations [26,27]. Second, pericardial fluid obtained at another hospital was not analyzed. Third, the CMR was performed at another hospital and only scanned film images were available for review. Therefore, no complete T2-weighted/STIR sequences could help assessing of pericardial edema. Last, the pathogenic contribution of the heterozygous *MEFV* E148Q variant could not be confirmed because neither family co-segregation analysis nor patient-derived functional assays were available.

## 4. Conclusions

In conclusion, this case exemplifies the shifting paradigm of recurrent pericarditis from an empiric, infection-centered model toward a mechanism-based autoinflammatory framework. It emphasizes the importance of recognizing IL-1–mediated pathways, reconsidering traditional diagnostic reflexes in TB-endemic settings, and acknowledging the real-world limitations that constrain optimal care. Integrating genetic insights into clinical decision-making holds significant promise for personalized therapy, but its full potential depends on effective targeted treatments become widely accessible.

## Figures and Tables

**Table 1:** Clinical features and treatment of three recurrent episodes of pericarditis and symptom-free intervals.

Results/Treatment	The First Recurrence	The Second Recurrence	The Third Recurrence	Symptom-Free Interval
CRP (mg/L)	247.4	97.5	160.1	<0.5–6.5
Pericardial effusion depth (mm)	23.5	4	12	0
Treatment before the Recurrence	None	Ibuprofen 300 mg bid; Colchicine intermittent dosing	Ibuprofen 200 mg qd; Colchicine intermittent dosing; Prednisone 5 mg qd	-
Intensified Treatment after Recurrence	Ibuprofen 300 mg bid; Colchicine 0.5 mg qd	Ibuprofen 600 mg q8h; Colchicine 0.5 mg bid; Prednisone 20 mg qd	Ibuprofen 200 mg bid; Colchicine 0.5 mg bid; Prednisone 5→2.5 mg qd	-
Symptom-free interval (weeks)	8	3	6	-

## Data Availability

Data are available upon request.
